# Editorial: Mechanisms and models of musculoskeletal pain and nonpharmacological treatment, volume II

**DOI:** 10.3389/fnint.2024.1476870

**Published:** 2024-08-26

**Authors:** William R. Reed, Maruti R. Gudavalli, Daniel F. Martins

**Affiliations:** ^1^Department of Physical Therapy, University of Alabama at Birmingham, Birmingham, AL, United States; ^2^College of Chiropractic Medicine, Keiser University, West Palm Beach Campus, Fort Lauderdale, FL, United States; ^3^Experimental Neuroscience Laboratory (LaNEx), Postgraduate Program in Health Sciences, University of Southern Santa Catarina - UNISUL, Palhoça, Brazil

**Keywords:** musculoskeletal, pain, nonpharmacological, manual therapy, integrative medicine, nonpharmaceutical, animal model

This Research Topic is a second volume highlighting the persistent need for more mechanistic-oriented research that investigates the underlying physiological responses to manual therapy and other nonpharmacological treatments for functional clinical improvements and pain management. Volume I of this Research Topic can be accessed here (https://www.frontiersin.org/research-topics/22475/mechanisms-and-models-of-musculoskeletal-pain-and-nonpharmacological-treatment/articles). The four articles included in Volume II are diverse (three clinical and one preclinical) and continue to illustrate the complexity of mechanistic research in the field of manual therapy and nonpharmacological treatment of musculoskeletal-related pain. Two articles in Volume II address assessment of peripheral inflammatory biomarkers following nonpharmacological interventions, while the remaining articles address either accurately measuring mechanical forces delivered during manual therapy treatment, or the clinical effects of combined core stability exercises and manual therapy on sacroiliac joint dysfunction.

The sole preclinical article (Dutra et al.) investigated the antihyperalgesic and anti-inflammatory effects of percutaneous vagus nerve electrical stimulation (pVNS) combined with exercise (swimming) in mice with hindpaw inflammation induced by injection of Freund's complete adjuvant. Mice were treated with either 30 min of swimming alone, or in combination with 10, 20, or 30 min of pVNS (via the auricular nerve branch in the left ear) over 4 consecutive days. Study outcomes included behavioral tests (i.e., edema, paw temperature, mechanical hyperalgesia) and changes in inflammatory cytokines (interleukin-6 [IL-6] and interleukin-10 [IL-10]) in the spinal cord and hindpaw tissues. It was found that 20 min of pVNS prolonged the mechanical antihyperalgesic effect for up to 2 h, while 30 min of pVNS prolonged this antihyperalgesic effect up to 7 h, and no effect of pVNS was demonstrated on either paw edema or paw temperature. While swimming by itself failed to alter IL-6 or IL-10 levels in the paw or spinal cord tissue, combined swimming and pVNS reduced IL-6 levels in both hindpaw and spinal cord tissues, and IL-10 in just the spinal cord. From this study, we see the benefit of combining nonpharmacological interventions (exercise and pVNS) on mechanical antihyperalgesia and the reduction of peripheral/central inflammatory cytokines. This study did not determine the precise physiological mechanisms responsible for these changes, but it thought that pVNS modulates activation of the HPA axis which influences the main organs of the immune system which synthesize pro-inflammatory cytokines, and/or that pVNS contributes to the production of pro-resolutive mediators such as resolverins and maresins. These findings contribute to a growing number of studies investigating the beneficial effects of combining of various types of exercise with pVNS to modulating pain-related inflammatory cytokines and other pain biomarkers for more effective clinical pain management.

In addition to the preclinical study investigating inflammatory cytokine modulation with the nonpharmalogical interventions of exercise and pVNS, Gevers-Montoro et al. investigated whether urinary levels of pro-inflammatory cytokine TNF-α could be beneficial in predicting clinical outcomes and/or characteristics of individuals with chronic primary low back pain (CPLBP). Changes in urinary TNF-α concentrations were compared between 24 CPLBP patients who underwent spinal manipulation treatment (eight visits) and asymptomatic age-matched controls. Concentrations of urinary TNF-α were elevated at baseline for the CPLBP group compared to asymptomatic controls, with patients with persistent CPLBP showing higher TNF-α levels than those experiencing episodic CPLBP. These findings suggest that urinary TNF-α concentrations may potentially be useful as a potential patient stratification biomarker to accurately discriminate between levels of CPLBP, however the limited subgroup sample size warrants caution with data interpretation. Pain intensity and the degree of disability were significantly reduced with spinal manipulation, however changes in TNF-α did not predict follow-up values in pain intensity, nor disability. As an observational study with a small sample size and lack of a control intervention group, study changes could not be attributed to the intervention or any other factors. However, future placebo-controlled studies will help determine the specific relationship between biomarker change, manual therapy delivery characteristics, and positive clinical outcomes.

Delivery characteristics of manual therapy was the topic of the third article (Siciliano et al.). Accurately measuring applied mechanical forces during nonpharmacological treatments such as spinal manipulation, joint mobilization, and massage is crucial to determining the potential relationship between manual therapy application/dosage and positive clinical outcomes. This article is a case report of a patient diagnosed with discogenic sciatica with a sequestered disc fragment at L5 and a motor deficit of the lower left extremity who underwent nonpharmacological treatment in the form of Cox Technique Flexion Distraction Decompression spinal manipulation, electrical muscle stimulation, infra-red light therapy, and a home exercise program. Force cells embedded within the instrumented treatment table accurately recorded bi-directional applied mechanical forces and motion data which then can be used to ensure objective treatment consistency and reproducibility by the treating clinician. Applied forces at specific flexion angles during treatment protocols were reported. The patient responded well to this nonsurgical treatment. The ability to accurately and reliably measure applied forces during manual therapy treatment may be used in future manual therapy studies to help determine the optimal delivery characteristics and positive clinical outcomes.

The fourth study (Yan et al.) investigated the effects of core stability exercises (CSE) and Mulligan's mobilization with movement (MWM) technique on sacroiliac joint dysfunction. Sacroiliac joint dysfunction is a frequent contributor to low back pain but is often misdiagnosed or treated inadequately. This study was a single-blind randomized controlled study involving 36 individuals divided into three groups (control, CSE, and CSE + MWM) and 18 intervention sessions. Pain and disability decreased with CSE and CSE + MWM compared to control. Significant decreases in lumbar flexion and left axial rotation range of motion occurred in the CSE group, while increases in lumbar extension and left lateral flexion range of motion occurred in the CSE+MWM group. Similar to the aforementioned preclinical study, this study suggests that combined exercise and nonpharmacological treatments may enhance functional and pain-related outcomes more than single interventions alone.

While there remains a great need for more preclinical and clinical nonpharmacologic mechanistic-oriented studies, studies that better define clinical outcomes as well as determine optimal nonpharmacological delivery characteristics will only serve to increase public acceptance and utilization of these interventions in the management of musculoskeletal pain.

